# The Potential Impact of Digital Biomarkers in Multiple Sclerosis in The Netherlands: An Early Health Technology Assessment of MS Sherpa

**DOI:** 10.3390/brainsci11101305

**Published:** 2021-09-30

**Authors:** Sonja Cloosterman, Inez Wijnands, Simone Huygens, Valérie Wester, Ka-Hoo Lam, Eva Strijbis, Bram den Teuling, Matthijs Versteegh

**Affiliations:** 1 Orikami Digital Health Products, Ridderstraat 29, 6511 TM Nijmegen, The Netherlands; inez@orikami.nl (I.W.); bram@orikami.nl (B.d.T.); 2 Institute for Medical Technology Assessment (iMTA), Erasmus University of Rotterdam, Burgemeester Oudlaan 50, 3062 PA Rotterdam, The Netherlands; huygens@imta.eur.nl (S.H.); wester@eshpm.eur.nl (V.W.); versteegh@imta.eur.nl (M.V.); 3 Erasmus School for Health Policy & Management, Erasmus University of Rotterdam, Burgemeester Oudlaan 50, 3062 PA Rotterdam, The Netherlands; 4 Department of Neurology, MS Center Amsterdam, Amsterdam University Medical Centers, Location VUmc, De Boelelaan, 1117 HV Amsterdam, The Netherlands; k.lam1@amsterdamumc.nl (K.-H.L.); e.strijbis@amsterdamumc.nl (E.S.)

**Keywords:** digital biomarkers, eHealth, digital health, AI, (early) Health Technology Assessment, multiple sclerosis, home monitoring, MS disease activity, MS disease progression, early detection, disease modelling, digital therapeutics

## Abstract

(1) *Background*: Monitoring of Multiple Sclerosis (MS) with eHealth interventions or digital biomarkers provides added value to the current care path. Evidence in the literature is currently scarce. MS sherpa is an eHealth intervention with digital biomarkers, aimed at monitoring symptom progression and disease activity. To show the added value of digital biomarker–based eHealth interventions to the MS care path, an early Health Technology Assessment (eHTA) was performed, with MS sherpa as an example, to assess the potential impact on treatment switches. (2) *Methods:* The eHTA was performed according to the Dutch guidelines for health economic evaluations. A decision analytic MS model was used to estimate the costs and benefits of MS standard care with and without use of MS sherpa, expressed in incremental cost-effectiveness ratios (ICERs) from both societal and health care perspectives. The efficacy of MS sherpa on early detection of active disease and the initiation of a treatment switch were modeled for a range of assumed efficacy (5%, 10%, 15%, 20%). (3) *Results*: From a societal perspective, for the efficacy of 15% or 20%, MS sherpa became dominant, which means cost-saving compared to the standard of care. MS sherpa is cost-effective in the 5% and 10% scenarios (ICERs EUR 14,535 and EUR 4069, respectively). From the health care perspective, all scenarios were cost-effective. Sensitivity analysis showed that increasing the efficacy of MS sherpa in detecting active disease early leading to treatment switches be the most impactful factor in the MS model. (4) *Conclusions*: The results indicate the potential of eHealth interventions to be cost-effective or even cost-saving in the MS care path. As such, digital biomarker–based eHealth interventions, like MS sherpa, are promising cost-effective solutions in optimizing MS disease management for people with MS, by detecting active disease early and helping neurologists in decisions on treatment switch.

## 1. Introduction

eHealth interventions play a growing role in shaping the future healthcare system. The integration of eHealth interventions can enhance the efficiency and quality of patient management and optimize the course of treatment for chronically ill patients [1] by alleviating pressure on health care systems when productivity of labor is restricted [2]. In this paper, we investigate the benefits of adding a digital biomarker–based eHealth intervention to the standard of care of multiple sclerosis (MS).

MS is the most prevalent chronic neurological disorder among young adults [3]. The severity and nature of symptoms and disability in MS depend on the location and extent of inflammatory demyelination and axonal loss in the central nervous system due to inflammation. Therefore, MS shows a highly individualized trajectory and large day-to-day variation [4]. Fatigue, decline in cognitive functions, impaired vision, motor and sensory deficits are the most common symptoms in persons with MS (pwMS) [4,5]. There is no cure for MS, but treatment is aimed at reducing neuroinflammation (and indirectly neurodegeneration) to prevent relapses and slow down disability progression. These disease modifying therapies (DMTs) are costly and the choice is plentiful. Current consensus recommends no evidence of disease activity (NEDA) as the treatment goal [6,7].

In the Netherlands, pwMS are under treatment by a neurologist, preferably complemented by a specialized MS nurse [8,9]. They usually have around one or two visits a year. Using MRI of the brain (and if necessary, the spinal cord), the presence of inflammatory disease activity is assessed, generally presenting as new or enlarged T2 lesions. The functioning of pwMS may be monitored by a variety of patient-reported (PRO) or performance-based outcome measures, such as test batteries to assess cognitive function and walking tests for ambulatory function for instance. The Expanded Disability Status Scale (EDSS) is the standard measure for how a person is affected by their MS. This combination of assessments of functioning, degree of disability and treatment effects, enable the determination of whether a pwMS is experiencing disease progression, a relapse or whether NEDA is maintained [6,7].

Typically, pwMS only remember certain days or periods that stand out, and the time in between is not recalled and the physician will not hear all information [10]. eHealth interventions and specifically those with objective measures, like digital biomarkers, in addition to PROs, can help monitor disease and symptom progression, and potentially disease activity [1,10].

Especially for persons with relapse-remitting MS, there are several treatment options and pwMS react differently to the different available drugs [6]. It is often a trade-off between the effectiveness of the drug and occurrence and severity of side effects, i.e., possibly overtreating or undertreating the patient. It is currently not possible to determine which treatment is the most appropriate for an individual pwMS. The disease course is highly heterogeneous and although we are able to assess the effectivity of a treatment according to NEDA [6], it is not yet possible to predict if and when pwMS will reach severe disability or secondary-progressive MS right at the moment after diagnosis. Additionally, subtle changes in functioning or symptoms and day-to-day variation are difficult to capture with the low frequent hospital visits that are currently the standard of care [6,7].

Because eHealth interventions can be applied in the home situation and this enables monitoring on a more frequent basis, the monitoring extends to the period between consultations and shows the individual course of symptoms. Therefore, the results can be used to detect disease activity early and find the optimal disease management for the individual patient.

### 1.1. eHealth Interventions in MS

Several eHealth interventions are currently developed and under investigation in MS [1]. These interventions support different aspects of the MS care path, like social, single use case, integrated and complex support, but with the common intention to improve the care path of pwMS leading to better outcomes. Social eHealth interventions (e.g., My Support Plus [11,12,13]) are usually meant for pwMS to get connected to other pwMS, to obtain information or to get in contact with their neurologist. Single use case solutions focus more on the disease and usually contain one or more measurement methods, which may be digital biomarkers or biomarker components. Scholz et al. [1] distinguish these interventions from the more integrated eHealth interventions, such as Floodlight (Genentech, Inc., Basel, Switzerland), MSCopilot (Ad Scientiam, Paris, France), MSPT (Cleveland Clinic Foundation & Biogen, Cleveland, OH, USA), etc. These digital biomarker–based eHealth interventions aim at enhancing MS monitoring and to better detect disease activity and progression so that better therapy can be applied.

**Figure 1:**
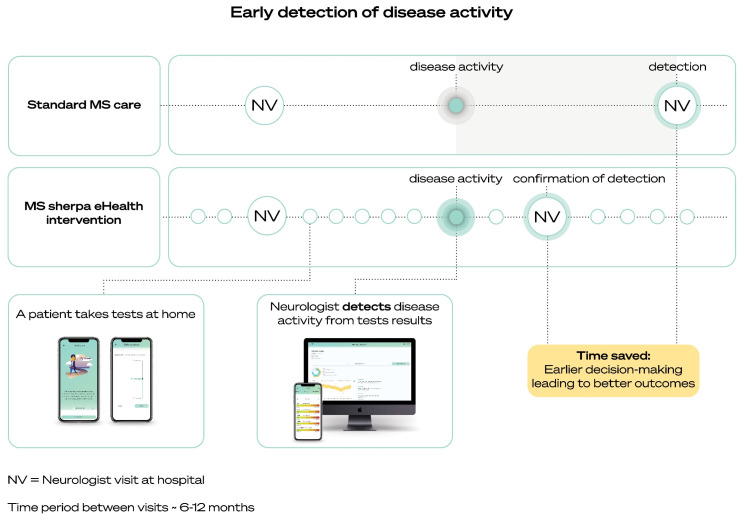
Graphical representation of the MS sherpa concept.

Another example of an integrated eHealth intervention containing digital biomarkers for MS, is MS sherpa (Orikami Digital Health Products, Nijmegen, The Netherlands). MS sherpa is a CE-certified eHealth intervention (medical device) intended to support the monitoring of persons with MS with the help of digital biomarkers, in order to give pwMS and their health care professionals personalized insight into the presence and progress of MS-related symptoms. The digital biomarkers are embedded in a smartphone application for pwMS and consist of tests that pwMS can perform regularly. The results are directly available for their neurologist via a web-based portal for caregivers, integrating MS sherpa into the MS care path. The Orikami Digital Biomarker platform on which the app and portal are built consists of several components to combine the sensors of the smartphone and user input with proprietary algorithms into digital biomarkers and of supporting modules such as a customer support, subscription and consent management and modules for regulatory compliance and authentication. A graphical presentation of MS sherpa concept is given in Figure 1.

The current MS sherpa digital biomarkers are validated to reliably measure cognitive processing speed and walking function [14,15,16]. These digital biomarkers represent relevant MS symptoms that are selected based on their relevance in MS and relation with disease activity and relapses. eHealth interventions require the willingness of the users to adhere to the intervention and to include the insights in disease management, therefore it is important to tailor the designs of eHealth interventions to the needs of the different users and to involve them in the development [17,18,19]. During the development of MS sherpa, input of different users, both pwMS and neurologists, were included via co-creation. Additionally, the designs have been tested via usability testing methods [18]. Adherence to eHealth interventions with digital biomarkers show promising results. MS Sherpa has shown in a one-month study that there was >90% adherence to the scheduled tasks [20]. This is in line with high adherence figures of other digital biomarker–based e-health interventions like Floodlight, which shows 70% adherence in a 24-week study [21], and an acceptability study with MSCopilot that shows that 85% of questioned pwMS are willing to use the intervention more than once a month and that 68% prefer the digital biomarkers over the MSFC [22], supporting the believe that adoption of and adherence to such interventions can be reasonably expected.

As eHealth interventions, and digital biomarkers more specifically, are a very nascent field, there are currently no RCTs that show their impact to personalized treatment. However, the potential impact of digital biomarker–based integrated eHealth interventions like MS sherpa in the MS care path is more and more being investigated in clinical trials. MS sherpa has multiple clinical trials in preparation or under investigation.

The Dutch Ministry of Health, Welfare and Sport and the iMTA institute aimed to show the potential impact of AI in healthcare, and MS sherpa was selected as a suitable eHealth intervention for Health Technology Assessment by both organizations, because of its already accumulated evidence and the availability of a model for MS to map the impact of an intervention on the care path.

### 1.2. (Early) Health Technology Assessment ((e)HTA)

To estimate the impact of health technologies, in terms of costs and benefits that fall upon the health care system and wider society, Health Technology Assessments (HTA) are conducted [23]. Cost-effectiveness analysis (CEA) is a central component of HTA. When a CEA is conducted before all effectiveness estimates have been collected in studies, the analysis is referred to as ‘early HTA’.

The result of an early HTA (eHTA) is an estimate of incremental costs and benefits with and without a new technology. The ratio between these increments gives the incremental cost-effectiveness ratio (ICER), which is compared with some reference value reflecting if the technology should be adopted in the basic benefit package [23]. Benefits are expressed in quality adjusted life years (QALYs). In the Netherlands, the National Health Care Institute determines the reference value for cost-effectiveness, based on the disease burden of the health care problem under study [24]. For MS, this value is set at EUR 50,000 per QALY [25].

In this article, we describe an eHTA analysis for the potential impact of MS sherpa, both from the societal and health care perspective using a recently published decision analytic model for MS treatments [25]. The analysis focused on the impact of MS sherpa on treatment decisions, and more specifically on switches of MS medication based on disease insights achieved with MS sherpa. The impact of digital biomarkers on treatment decisions is one of the important concepts to be tested for the MS field.

## 2. Materials and Methods

### 2.1. Early Health Technology Assesment (HTA), Concept and Analyses

This eHTA was performed according to the Dutch guidelines for economic evaluations [26]. The MS model was used to estimate the costs and benefits of MS standard care with and without the use of MS sherpa and expressed in an ICER.

ICER = (costs new intervention − costs standard care)/(health gains new intervention − health gains  standard care) = € per QALY

This ICER is compared to the reference value for the maximum costs that the society is willing to pay for 1 additional QALY within MS in the Netherlands [25].

These calculations were performed from both a societal and a health care perspective. In a societal perspective, all relevant costs and benefits related to MS are included in the cost-effectiveness analysis (CEA), regardless of who bears the costs or enjoys the benefits. The costs of an intervention are therefore not limited to costs within health care, but costs outside health care are also included in the CEA. Costs outside of health care include the costs of informal caregivers and reduced productivity in paid and unpaid work due to health problems. This is the standard perspective as prescribed by the National Health Care Institute [26]. In a health care perspective, costs outside healthcare (i.e., costs of informal care and productivity losses) are not included.

### 2.2. MS Model and Clinical, Costs and Quality of Life Input

**Figure 2:**
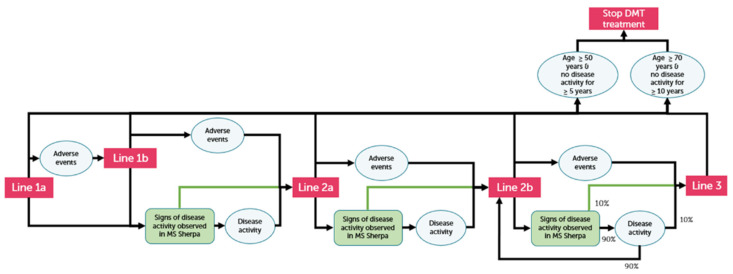
Schematic representation of the MS model.

The CEA was performed using the MS model developed by Huygens and Versteegh [25]. This model describes the lifetime of a pwMS based on MS relapses (Annual Relapse Rate (ARR)) and MS disease progression (EDSS). The rate of progression and relapses are influenced by the efficacy of the MS medication. Treatment switching was allowed for up to five lines of treatment. Besides disease progression and relapse, adverse events of MS medication, the option of discontinuing MS medication, health-related quality of life (HRQoL) and mortality rate were included in the model. All costs related to the treatment of MS (MS medication, other health care costs, informal care, productivity loss) were included in the model. During the development of this model, neurologists were involved to confirm clinical assumptions and decisions in this model. A graphical representation of the model is given in Figure 2.

The output of the MS model are the lifetime costs and subsequent the lifetime clinical MS outcomes (‘benefits’) represented as EDSS and ARR for pwMS, both for MS care with and without MS sherpa. These modeled costs and benefits are used for the CEA and ICER calculations.

To assess uncertainty of the model and tested intervention, univariate sensitivity analyses were performed, in which the value of one key parameter at a time is changed into a higher and lower value than assumed in the base-case analysis. The results of this analysis, which will be presented in a tornado diagram, give insight in which parameters have the most impact on the cost-effectiveness of MS sherpa. This might serve as input for future improvements of MS sherpa or for other eHealth interventions that share the same or equivalent components or digital biomarkers. The cost-effectiveness in this analysis is presented as the net health benefit, calculated with the following formula:

Net Health Benefit = Total QALYs − (total costs/Cost-effectiveness threshold (€50,000/QALY))

### 2.3. MS Sherpa and Potential Effects

MS sherpa is a CE Class I Medical Device under MDD consisting of a smartphone application for pwMS and a web-based portal for caregivers. For this eHTA, MS sherpa version 1.12 was used. The MS sherpa 1.12 app for pwMS contains two digital biomarkers: one as an indicator for cognitive processing speed, an adaptation of the Symbol Digit Modalities Test (SDMT); and one for walking speed, an adaptation of the two-minute walking test (2MWT). Both are smartphone adaptations of standardized tests that assess important symptoms of MS and are suitable for frequent self-administration. The SDMT was chosen because of its sensitivity to changes in mental status during clinical relapses, and during isolated cognitive relapses without changes on EDSS [27,28,29]. The 2MWT was chosen because of its strong correlation with EDSS [30]. Both digital biomarkers showed robust concurrent validity and test–retest reliability [14,15,16], and for the SDMT also the construct validity was shown by distinguishing pwMS from healthy controls [15]. As such, these digital biomarkers are reliable tools for monitoring relevant MS symptoms, enabling pwMS to monitor their symptoms objectively and more frequently in their home situation.

In addition to the objective measurements, pwMS can answer a daily questionnaire in MS sherpa, containing several Likert-scale questions on fatigue, pain, stress, memory, concentration and the impact of MS on the day. PwMS can also leave notes in the app on events and symptoms as deemed relevant by the patients. The clinician portal contains a dashboard for caregivers that shows their pwMS’ results from the MS sherpa app. They can see the SDMT and 2MWT scores as individual data points, but also informative curves over time using multiple measurements to model individualized performance trajectories. This is giving more detailed insights in MS symptom changes over time than during clinical visits. Recent results [31] showed that more frequent monitoring in combination with smart algorithms significantly reduces signal-to-noise ratio of the measurements and the ability to follow individualized trajectories (patent pending: N2028255). For the MS field these innovations make it possible to detect more subtle changes with the potential to detect disease progression and relapses earlier. Moreover, the answers to the questionnaire and the notes that pwMS write can also be viewed, giving context to the measurements. This can improve the shared decision-making process between the neurologist and pwMS. The information from MS sherpa can help tailor the care path to the individual pwMS, resulting in earlier treatment switches to other, more effective treatment.

The MS sherpa solution and its effect on treatment decisions was operationalized in the MS model as shown in Figure 2: with the use and insights of MS sherpa, pwMS and neurologists will have insight into active disease sooner and, as a consequence, will switch to the next treatment line earlier than without MS sherpa. Second line treatments are generally considered to be more effective, but also more expensive treatments. Timely switches to these treatments could prevent disease progression and relapses, which will subsequently lead to health and quality of life benefits and potential cost savings. It is assumed that all pwMS with an EDSS below 7 will use the MS sherpa app, as pwMS with an EDSS of 7 and higher are wheelchair-dependent and not able to use the app in the intended way [32]. As the effect of MS sherpa on treatment switches is not yet known and is dependent of the efficacy in the detection of disease activity, different assumptions of the efficacy of MS sherpa were tested in the eHTA. MS sherpa’s efficacy is defined as the proportion of pwMS who are detected early by MS sherpa to have disease progression or relapse that will switch to a next line treatment prior to that disease progression or relapse occurring. Four scenarios for MS sherpa’s efficacy in detecting disease activity earlier than standard care were chosen: 5, 10, 15 and 20 percent. As the effect size of MS sherpa is not yet known, and effects of comparable eHealth interventions are also not available, the effect sizes were discussed with neurologists who are involved in the ongoing clinical studies with MS sherpa. They are familiar with the insights provided by MS sherpa and the role of the digital biomarkers in MS sherpa, which are smartphone adaptations of commonly used clinical outcome measures (i.e., SDMT and 2MWT). They indicated that it is currently difficult to estimate the efficacy of MS sherpa for early detection of disease activity, but they confirmed that the chosen range seems plausible given the current development stage of MS sherpa.

The cost of MS sherpa is currently estimated to be EUR 480 per patient per year.

## 3. Results

### 3.1. Clinical Effects (Benefits) of MS Sherpa (Modeled)

**Figure 3:**
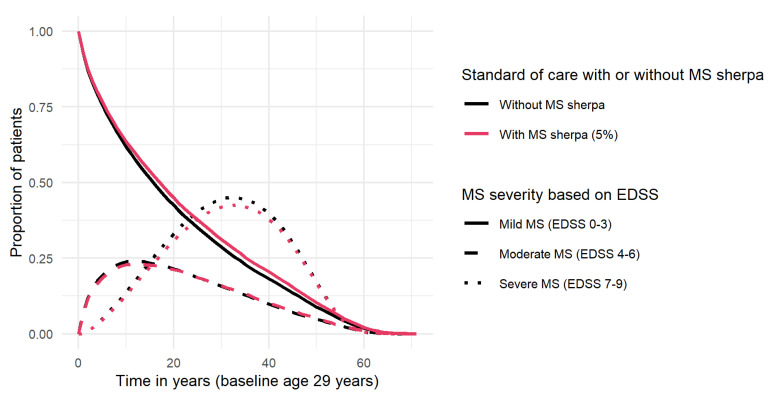
Progression in MS severity over time measured with EDSS over time, with and without use of MS Sherpa.

**Figure 4:**
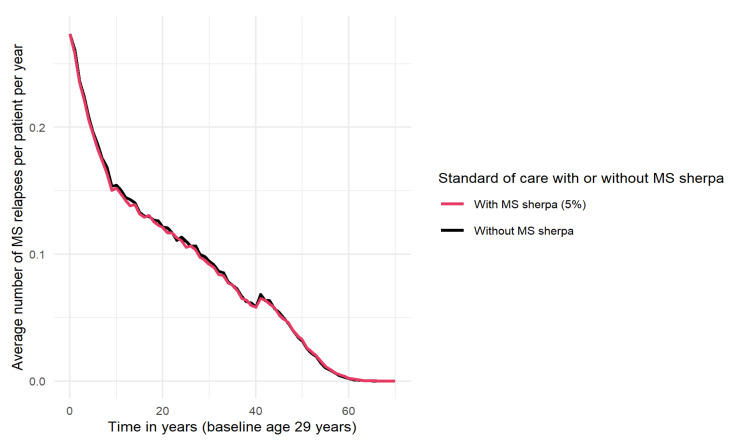
Average number of MS relapses per patient per year over time, with and without use of MS sherpa. The hiccup at t = 41 can be explained by the rule in the MS model (see Figure 2) that pwMS will discontinue DMTs at age 70 in the absence of disease activity ≥10 years and the risk of relapses is not reduced by the DMT anymore.

Based on the MS model, the clinical outcomes for MS care both with and without MS sherpa were modeled. Figure 3 and Figure 4 demonstrate the reduction in disease progression and relapse rates of MS sherpa use, under the assumption of 5% efficacy. Figure 3 shows that, under this assumption, the use of MS sherpa slows down disease progression: a larger proportion of pwMS have mild disability due to MS (EDSS 0–3) for a longer period of time, a smaller proportion of pwMS develop severe disability (EDSS 7–9), and severe disability develops later than without the use of MS sherpa. For example, 10 years after the diagnosis of MS, the proportion of pwMS who still have mild disability due to MS is higher with use of MS sherpa (63.5%) compared to without (61.7%). The number of pwMS who had progressed to moderate disability 10 years after MS diagnosis is lower with the use of MS sherpa (22.8%) than without the use of MS sherpa (23.4%). The same is true for the percentage of pwMS with severe disability: 13.0% with MS sherpa compared to 14.2%, without MS sherpa.

In addition, Figure 4 shows that the ARR is slightly lower among users of MS sherpa. Ten years after the diagnosis of MS, the probability of an MS relapse without the use of MS sherpa is 15.7% compared to 15.3% with the use of MS sherpa.

### 3.2. Cost-Effectiveness of MS Sherpa, Societal Perspective

The cost-effectiveness results for MS sherpa compared to standard of care without MS sherpa for each of the MS sherpa efficacy scenarios from a societal perspective are shown in Table 1. When assuming 5% and 10% efficacy of MS sherpa, QALYS were gained (0.43 and 0.87, respectively), but this was associated with higher costs. Compared to the reference value of EUR 50,000 per QALY, MS sherpa is cost-effective in these scenarios. In the scenarios where the assumed effect of MS sherpa was 15% or 20%, MS sherpa became dominant, which means costs are saved while pwMS yielded 1.33 or 1.78 additional QALYs.

### 3.3. Cost-Effectiveness of MS Sherpa, Health Care Perspective

Table 2 presents the cost-effectiveness results from a health care perspective (i.e., without costs of informal care and productivity loss). The results show that total costs are lower, but that the difference in costs between standard of care with and without MS sherpa is larger because some of the benefits of using MS sherpa, such as reducing informal care and productivity loss due to less disease progression and relapses, are no longer included in the cost calculations. Nevertheless, the ICER is still below the reference value of EUR 50,000.

### 3.4. Sensitivity Analysis, Tornado Diagram

**Figure 5:**
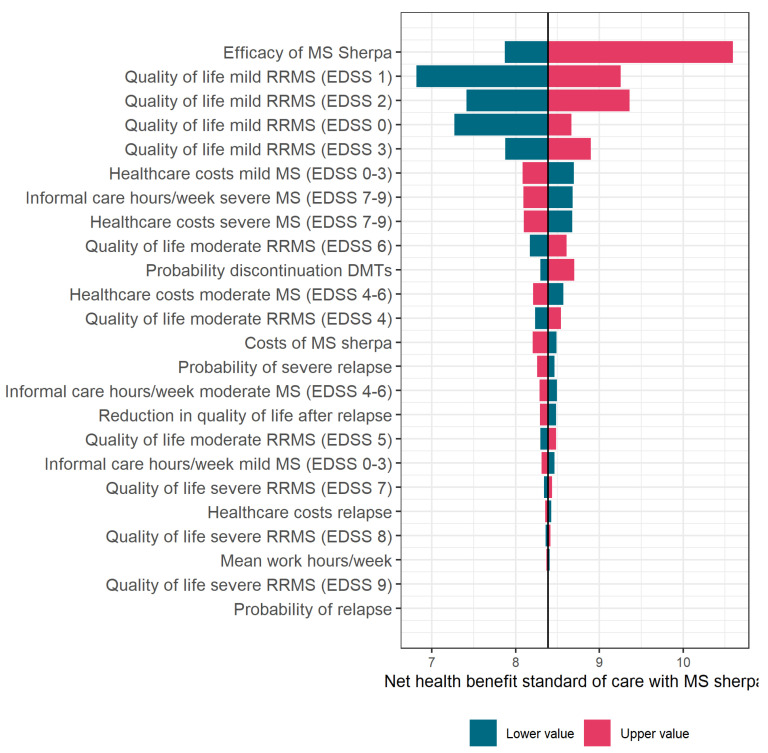
Tornado diagram with results of the univariate sensitivity analysis.

The results of the univariate sensitivity analyses are shown in Figure 5. The vertical line represents the net health benefit (this is the number of QALYs reduced with the total costs, in which the QALY has a value of EUR 50,000) in the base-case analysis (i.e., 5% efficacy of MS sherpa). The bars represent the impact of the different parameters on the cost-effectiveness results.

These results show that the assumed effect of MS sherpa has substantial impact on the net health benefit. The higher the efficacy of MS sherpa in detecting disease activity, the higher the effect on treatment switches and the higher the net health benefit. This means that it would be worthwhile to focus on further improving the efficacy of MS sherpa. In addition, the diagram shows that quality of life and health care costs of pwMS with mild MS (EDSS 0–3) have substantial impact on the net health benefit. This is not surprising as pwMS spend a large part of their life with mild MS, and this period is prolonged when using MS sherpa.

All other key parameters showed to have less influence on the outcomes of the model when assuming a 5% efficacy of MS sherpa. For instance, when the annual costs of MS sherpa per patient were increased to EUR 1000, even with 5% efficacy, it is still cost-effective.

## 4. Discussion

### 4.1. Principal Results and Implications for Clinical Practice and MS Society

MS sherpa is an eHealth intervention aimed at enabling (home) monitoring of pwMS with the help of digital biomarkers, in order to give pwMS and their caregivers individual insights into the presence and progress of MS-related symptoms and disease activity. In this research, we modeled how with the insights from digital biomarker interventions like MS sherpa (efficacy), neurologists together with pwMS have the potential to decide earlier to switch to more effective MS medication, with the intention to prevent or slow down disability worsening and disease progression. The recent Huygens and Versteegh MS model [25] that simulates the disease progress over the lifetime of a patient was used to show whether using MS sherpa to support treatment decisions would be cost-effective. The eHTA showed that under all efficacy assumptions MS sherpa is cost-effective from both a societal and health care perspective in the MS care path. Moreover, in the societal perspective MS sherpa can become dominant and cost saving when the efficacy of detecting disease activity early is 15% or higher and higher proportions of pwMS switch medication.

The eHTA as performed in this research gives valuable insight into the potential cost and benefits of digital biomarkers in MS and supports the use of new solutions like MS sherpa by neurologists to detect early symptom progression and disease activity of pwMS. While the effect of a digital biomarker–based eHealth intervention on clinical outcome can seem moderate from this analysis, it should be placed in the right context. First of all, the effect on clinical outcomes can be strong for an individual pwMS where preventing one relapsewith the associated brain damage can mean the difference between years with or without work or physical dysfunction on the long term. Second, cost-effectiveness of current DMTs has long been debated and health gains come at a high costs. For example, it was shown that cost-effectiveness of MS DMTs in the US far exceeded USD 800,000/QALY [33]. The results of the current analysis on an ICER of EUR 14,535/QALY gives another dimension to more appropriate investment and reimbursement decisions. At this moment, the literature is lacking a strong benchmark of cost-effectiveness of monitoring solutions in MS. It would be relevant for future research to show how self-monitoring with digital biomarker–based eHealth interventions benchmarks with other monitoring solutions like MRI, test batteries administered by a clinician and recent blood-based biomarkers.

The presented eHTA was performed both from a societal and health care perspective. As MS usually onsets in early adult life, when persons are still active and taking part in the working life, developing MS will affect not only health care costs but especially also the non-health care costs like employability. Therefore, we feel that the presented eHTA with a societal perspective is the most comprehensive. The health care perspective is of importance for hospitals or health insurance companies in adopting digital biomarker–based eHealth interventions and gives them more insights where costs and benefits are falling within the health care setting. Reimbursement decisions based on health care perspective alone might not be appropriate for eHealth interventions; therefore, this confirms that it is advisable to include the societal perspective in reimbursement models for such interventions.

### 4.2. Relation to Previous Work

The efficacy of MS sherpa in detecting disease activity and enable optimal disease management earlier compared to standard care, is now varied between 5% and 20%. Digital biomarker–based eHealth interventions for monitoring MS, like MS sherpa, are relatively new in the MS field. The current literature gives us no guidance in the potential clinical impact of these solutions. A benchmark for integrated eHealth interventions and digital biomarkers on MS outcomes is not (yet) available. As explained, the impact of MS sherpa insights on treatment decisions are thought to achieve at least the assumed efficacy of 5%, based on the described evidence and setting. Additionally, a minimum efficacy of 5% as a starting point seems plausible according to interviewed neurologists involved in testing the MS Sherpa solution.

The MS model was used to calculate the costs and clinical benefits, and was used in the eHTA. While all models require assumptions, this model is shown to be a good predictor for short-term switch behavior when validated against external data [25]. Moreover, this is the first model that takes into account subsequent medication steps, as a complete treatment sequence cost-utility model for MS. As the presented concept of MS sherpa is mainly focused on treatment decisions, this MS model seems currently the best basis to test the potential effects of an eHealth intervention like MS sherpa. As such, we believe that the chosen model is both fit for purpose and state-of-the-art in showing impact by treatment decisions on switching medication.

### 4.3. Considerations and Limitations

Next to the abovementioned assumptions in the model, some aspects can still be considered. Firstly, in the effectiveness of MS sherpa on treatment switches, the entire MS population with an EDSS below 7 is included. The adoption rate of an eHealth intervention might be a smaller proportion of this population. Moreover, subgroups may be identified in which a higher or lower effect is to be expected; for instance, neurologists may be able to identify pwMS for whom a higher gain from using MS sherpa is expected.

Secondly, the MS model is based on the MS care path in the Netherlands and the CEA is based on guidelines from the National Health Care Institute [26], which may not be applicable in other countries. Naturally, changes in the MS field in the future (e.g., new treatment options become available) can also influence the underlying assumptions in the MS model.

Besides the efficacy of MS sherpa in the MS care path, adherence to and acceptance of these kind of interventions is also important. Using eHealth interventions during a lifetime (or until EDSS is 7 or higher as modeled) might be challenging, which might reduce its impact. Usually, pwMS show their first symptoms at the age of 20 to 40 years; consequently, they live with this chronic disease for several decades, which is why these patients may be important early adopters of emerging eHealth trends [1,34]. A part of the pwMS indicated that using eHealth tools confront them with their disease. On the other hand, pwMS that start using MS sherpa showed high adherence to scheduled tests and valued the insights [20].

Next to the adherence of pwMS to the eHealth intervention, the adoption of MS sherpa by neurologists and its use in treatment decisions is an important factor [20]. The current model assumes that the efficacy of MS sherpa is directly related to treatment switch. It is expected that neurologists perform additional clinical assessments before switching treatment. Growing evidence, improved user experience, training of clinicians and algorithm improvements will help tackle this challenge.

### 4.4. Further Research

The key assumption that should be further investigated is the efficacy of the MS sherpa intervention in detecting disease activity early. Especially a clinical study that determines the sensitivity and specificity of the intervention in early detection of disease activity compared to standard care would be valuable. A multi-center RCT with MS sherpa as integrated eHealth intervention is scheduled presently.

In the meantime, the eHTA results show that by increasing the effect of MS sherpa on treatment switches, more benefits could be gained and as such the ICER becomes more favorable for MS sherpa. Increasing the MS sherpa efficacy is shown to be the most sensitive parameter of the model and therefore confirms that this should be the main focus in further development. As there are no univocal criteria for reimbursement of eHealth interventions in current reimbursement models, the sensitivity analyses provide us with helpful insights into which aspects of the eHealth intervention to focus on. Improving the efficacy can be achieved by improving the algorithms in the MS sherpa tool or adding measurements, so that disease progression, subclinical disease activity and relapses can be detected or predicted earlier.

Besides earlier detection of disease activity and subsequent treatment switches, MS sherpa potentially has other benefits within the MS care path, like supporting stopping of MS treatment in stable pwMS, earlier diagnosis of SPMS, improved self-efficacy and patient empowerment, monitoring effects of therapies other than DMTs, etc. The use of eHealth interventions might substitute clinical procedures with home/remote testing, leading to cost and efficiency gains. Especially for pwMS, not only disease outcomes are important, but self-efficacy and patient empowerment might be more relevant drivers for them to adopt eHealth interventions like MS sherpa [20]. Future research will also focus on a broader spectrum of benefits than the impact on earlier treatment switch alone.

## 5. Conclusions

eHealth interventions hold the promise of alleviating pressure on the health care labor force and improve the lives of patients. Several eHealth initiatives are underway in the MS field, but the evidence on their impact on pwMS, MS care path and wider society is still lacking. Digital biomarker interventions for home-monitoring of pwMS like MS sherpa are promising. This research showed positive impact from using a digital biomarker–based eHealth intervention for early detection of active disease and switching treatment accordingly. This eHTA for MS sherpa is the first to combine a complex decision analytical model which captures lifetime treatment sequences with an MS-specific eHealth intervention. The results indicate the potential of MS sherpa to be cost-effective or even cost-saving. It is shown that its use may increase costs within the health care setting, but that these costs are offset by savings outside the health care setting. Dependent on the efficacy of the solution in early detection of active disease, MS sherpa has the potential to become dominant. The results of future and ongoing research should validate the assumptions on efficacy of MS sherpa incorporated in the model. Moreover, improving MS sherpa may further increase benefits.

## Figures and Tables

**Table 1:** Cost-effectiveness results of MS sherpa versus standard care from a societal perspective.

	Total		Difference between Standard Care and MS Sherpa		
Scenario	Costs	QALYs	Costs	QALYs	ICER
MS standard Care	€614,732	20.51			
MS sherpa 5%	€620,990	20.94	€6258	0.43	€14,535
MS sherpa 10%	€618,288	21.38	€3556	0.87	€4069
MS sherpa 15%	€614,538	21.84	€−194	1.33	D
MS sherpa 20%	€611,073	22.29	€−3659	1.78	D

QALY = Quality Adjusted Life Year. D = Dominant (lower costs and more benefits).

**Table 2:** Cost-effectiveness results of MS sherpa versus standard care from a health care perspective.

	Total		Difference between Standard Care and MS Sherpa		
Scenario	Costs	QALYs	Costs	QALYs	ICER
MS Standard Care	€540,345	20.51			
MS sherpa 5%	€539,528	20.94	€9183	0.43	€21,328
MS sherpa 10%	€539,803	21.38	€9458	0.87	€10,822
MS sherpa 15%	€539,101	21.84	€8756	1.33	€6574
MS sherpa 20%	€538,703	22.29	€8358	1.78	€4696

## Data Availability

The data presented in this study are available in Huygens & Versteegh. Modelling the cost-utility of treatment sequences for multiple sclerosis. Value in Health. Accepted for publication May 2021.
